# The genomic potential of photosynthesis in piconanoplankton is functionally redundant but taxonomically structured at a global scale

**DOI:** 10.1126/sciadv.adl0534

**Published:** 2024-08-16

**Authors:** Alexandre Schickele, Pavla Debeljak, Sakina-Dorothée Ayata, Lucie Bittner, Eric Pelletier, Lionel Guidi, Jean-Olivier Irisson

**Affiliations:** ^1^ Sorbonne Université, CNRS, Laboratoire d’Océanographie de Villefranche, LOV, F-06230 Villefranche-sur-Mer, France.; ^2^ Sorbonne Université, Muséum National d’Histoire Naturelle, CNRS, EPHE, Université des Antilles, Institut de Systématique, Evolution, Biodiversité (ISYEB), F-75005, Paris, France.; ^3^ SupBiotech, Villejuif, France.; ^4^ Sorbonne Université, CNRS, IRD, MNHN, Laboratoire d’Océanographie et du Climat, Institut Pierre Simon Laplace, LOCEAN-IPSL, F-75005 Paris, France.; ^5^ Institut Universitaire de France, Paris, France.; ^6^ Metabolic Genomics, Genoscope, Institut de Biologie François Jacob, CEA, CNRS, Université d'Evry, Université Paris Saclay, 91000 Evry, France.; ^7^ Research Federation for the Study of Global Ocean Systems Ecology and Evolution, FR2022/*Tara* Oceans GOSEE, Paris, France.

## Abstract

Carbon fixation is a key metabolic function shaping marine life, but the underlying taxonomic and functional diversity involved is only partially understood. Using metagenomic resources targeted at marine piconanoplankton, we provide a reproducible machine learning framework to derive the potential biogeography of genomic functions through the multi-output regression of gene read counts on environmental climatologies. Leveraging the Marine Atlas of *Tara* Oceans Unigenes, we investigate the genomic potential of primary production in the global ocean. The latter is performed by ribulose-1,5-bisphosphate carboxylase/oxygenase (RUBISCO) and is often associated with carbon concentration mechanisms in piconanoplankton, major marine unicellular photosynthetic organisms. We show that the genomic potential supporting C_4_ enzymes and RUBISCO exhibits strong functional redundancy and important affinity toward tropical oligotrophic waters. This redundancy is taxonomically structured by the dominance of Mamiellophyceae and Prymnesiophyceae in mid and high latitudes. These findings enhance our understanding of the relationship between functional and taxonomic diversity of microorganisms and environmental drivers of key biogeochemical cycles.

## Teaser

Metagenomic data and machine learning advances reveal spatial redundancy in the genomic potential for tropical ocean C_4_ enzymes.

## INTRODUCTION

Marine carbon fixation is largely performed by the piconanoplankton, responsible for 30 to 50% of global primary production (*1*, *2*). Piconanoplankton encompasses the unicellular eukaryotic marine plankton from the lower nano- to pico-size fractions (0.8 to 5 μm; also referred to as nano- and picoeukaryotes), including small diatoms, dinoflagellates, or prymnesiophytes. We hereafter refer to as piconanoplankton, following the *Tara* Oceans size fractions (*sensu 3*). These organisms are among the most diverse and abundant in the sunlit layer of the world ocean (*3*–*5*). In nutrient-poor areas, such as the oligotrophic open ocean, they locally contribute up to 80% of the phytoplanktonic biomass (*6*).

**Fig. 1.**
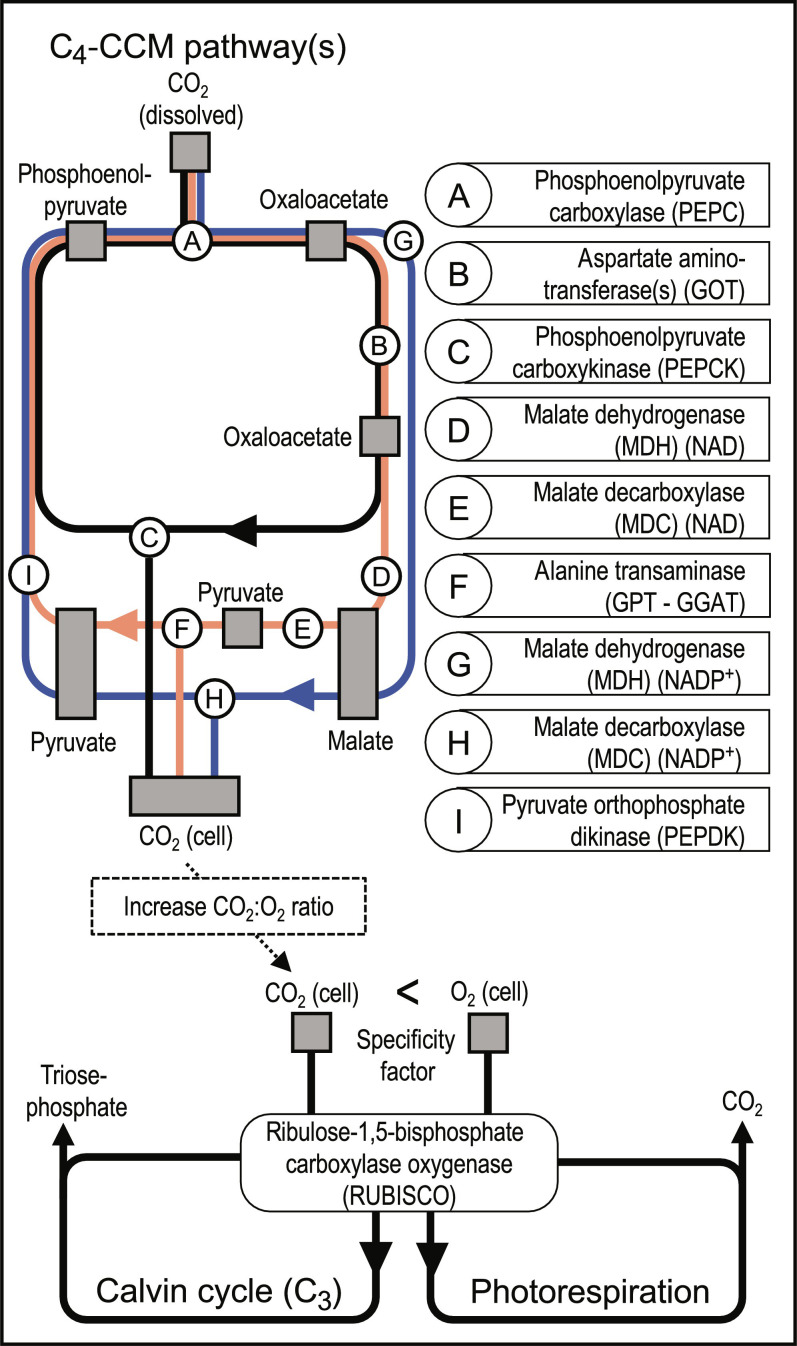
Diagrammatic representation of the main enzymes and metabolites participating in the C_4_ carbon concentration mechanisms, C_3_ Calvin cycle, and photorespiration. Note that subcellular compartment and secondary metabolites are not represented. Enzyme names follow the Kyoto Encyclopedia of Genes and Genomes terminology. The three main currently described acid decarboxylation types are represented in blue [malate decarboxylase–nicotinamide adenine dinucleotide phosphate (MDC-NADP)], orange [MDC–nicotinamide adenine dinucleotide (MDC-NAD)], and black [phosphoenolpyruvate carboxykinase (PEPCK)], respectively.

Most of the photosynthetic production on Earth relies on the ribulose-1,5-bisphosphate carboxylase/oxygenase [RUBISCO; (*7*)]. This enzyme is also responsible for photorespiration (Fig. 1). The latter is an energetically costly and metabolically inefficient pathway that consumes O_2_ to produce CO_2_ (*8*). However, RUBISCO does not clearly discriminate between CO_2_ and O_2_. RUBISCO emerged ~2 billion years ago in a period characterized by low oxygen (*9*). Therefore, its carboxylase function is unexpectedly inefficient relative to its oxygenase function, when considering the contemporary CO_2_:O_2_ ratio (*10*). The affinity of the carboxylase function relative to the oxygenase function of RUBISCO is referred to as the specificity factor (Fig. 1) that is variable across the tree of life, including marine phytoplankton (*9*, *11*). To compensate for the relative inefficiency of the carboxylase function of RUBISCO, carbon fixation pathways evolved ~30 million years ago when atmospheric CO_2_ levels were estimated under 200 parts per million (ppm) (*12*, *13*). This induced selective pressure toward higher carbon fixation efficiency and led to the emergence of RUBISCO of higher specificity factor and various carbon concentration mechanisms (CCMs; i.e., biophysical or biochemical mechanisms). The latter aims to compensate for the specificity factor of RUBISCO by concentrating CO_2_ at its active site (*8*).

Among biochemical CCMs, C_4_ enzymes independently evolved across a large variety of marine and terrestrial lineages (*8*, *14*). The C_4_ cycle is performed through three acid decarboxylation types (Fig. 1), all leading to an increase of the CO_2_:O_2_ ratio at the active site of RUBISCO (*15*): the malate decarboxylase–nicotinamide adenine dinucleotide phosphate (MDC-NADP) type, the MDC–nicotinamide adenine dinucleotide (MDC-NAD) type, and the phosphoenolpyruvate carboxykinase (PEPCK) type. The common enzyme to all C_4_ acid decarboxylation types is phosphoenolpyruvate carboxylase (PEPC), fixing CO_2_ in the cytosol by producing oxaloacetate (Fig. 1). In the MDC-NADP type, oxaloacetate is transferred to the chloroplast and reduced to malate. The latter is then decarboxylated, producing CO_2_ and pyruvate, which is converted back to phosphoenolpyruvate (Fig. 1, blue pathway). In the MDC-NAD type, oxaloacetate is transferred to the mitochondria and reduced to malate. The decarboxylation reaction transfers CO_2_ to the chloroplast by producing pyruvate that is transferred back to the chloroplast to be converted to phosphoenolpyruvate (Fig. 1, orange pathway). Last, the PEPCK type directly converts the mitochondrial oxaloacetate to phosphoenolpyruvate (Fig. 1, black pathway). However, it partially performs the MDH-NAD reduction and MDC-NADP decarboxylation reactions to balance the adenosine 5′-triphosphate (ATP) and NADPH budget, leading to common reactions and enzymes between acid decarboxylation types (*15*). In the terrestrial realm, both physiological measurements and stable isotope techniques confirmed the presence of C_3_ photosynthesis across a large range of environmental conditions, conversely to C_4_ photosynthesis that is adapted to warm, nutrient-poor, and high irradiance conditions (*12*, *16*). In the marine realm, however, only a few studies explored the environmental affinity of C_4_ photosynthesis regarding terrestrial-based hypotheses [see, e.g., (*13*, *14*, *17*)]. The potential for C_4_ photosynthesis is highly suspected in key piconanoplankton lineages such as Mamiellophyceae and Prymnesiophyceae. Currently, subcellular evidence for C_4_ enzymes include (i) MDC-NADP and PEPC in *Ostreococcus Tauri* (*18*); (ii) MDC-NADP, PEPC, three different oxoglutarate-to-malate translocator and pyruvate phosphate dikinase (PEPDK) in various *Micromonas* strains (*19*); and (iii) PEPC in the Prymnesiophyte *Emiliania huxleyi* [plastid presence and gene encoding (*20*)]. However, because of their small size (i.e., 0.8 to 5 μm) and poor representation in culture collections (*21*), physiological measurements and stable isotope analysis are lacking for natural piconanoplankton populations. Therefore, the genomic potential supporting C_3_, and C_4_ photosynthesis and its associated biogeography and functioning remains scarcely documented (*13*, *14*, *16*).

Recent global expeditions focusing on surface plankton sampling, together with advances in metagenomic sequencing, provided unique data to address the genomic potential and biogeography-related gaps [see, e.g., (*22*–*25*)]. In this context, metagenomics data are of growing interest to explore the hidden taxonomic and functional diversity potentially related to carbon fixation in piconanoplankton [see, e.g., (*26*, *27*)]. For example, genome-resolved metagenomics (*28*) based on the *Tara* Oceans eukaryotic metagenome led to the reconstruction of ~800 metagenome-assembled genomes [MAGs; (*29*)]. The latter are defined as genome-based taxonomic units, functionally and taxonomically annotated, and quantified by their associated genome-wide metagenomic reads. Therefore, MAGs offer the unique opportunity to study the genomic potential supporting carbon fixation and its biogeography, through both a functional and a taxonomic prism.

Habitat modeling is a popular niche theory–based tool to estimate species’ biogeography according to the environmental conditions in which they are observed (*30*). Marine organisms are known for their important sensitivity to their surrounding environmental conditions, influencing growth, reproduction, and metabolic efficiency across all life stages (*31*). Thus, habitat modeling has been widely used to project the past, present, and future biogeography across various marine organisms, from zooplankton to fishes and marine mammals [see, e.g., (*32*)]. However, omics-based habitat modeling is still an emerging field to explore functional and taxonomic biogeography associated with unicellular planktonic organisms (*33*–*35*). Building on the abovementioned properties associated with MAGs, habitat modeling is transferable to genomic potential, thus exploring the quantitative response of the associated taxonomic and functional gene annotations to environmental conditions.

Here, complementing recent studies focusing on prokaryote or eukaryote-environment relationships (*26*, *33*, *34*), we provide an original, machine learning–based, comprehensive, and reproducible framework to derive the biogeography of the genomic potential related to metabolic functions, from metagenomic-based relative abundances data. Using multivariate boosted tree regressors [MBTRs; (*36*)], we simultaneously project the biogeography of selected genomic functional annotations while accounting both for their interactions and environmental responses. We applied this framework to metagenome-based protein functional clusters (PFCs; hereafter referred to as “clusters”) linked to RUBISCO and C_4_ enzymes only, in marine piconanoplankton. Compared to a more traditional approach (i.e., searching reads in a functional database using sequence similarity), our methodology combining MAGs and PFCs offers several advantages. The quantitative signal resulting from a MAG is (i) standardized by the genome length and (ii) corresponds to a taxonomic identity. Combined with PFCs, (iii) it also includes the fraction of signal corresponding to not yet annotated genes. Thus, this approach offers a more robust quantitative framework than traditional approaches, representative of eukaryotic plankton diversity in open oceans [39.1 billion reads recruited, ~97% identity, ~25 giga–base pair (Gbp) (*29*)] and transferable to a variety of functions or enzymes of interest using the already computed PFC network. Last, habitat modeling provides an interesting tool to estimate the response and co-dominance patterns of C_4_ enzymes and RUBISCO to environmental conditions representative of the global ocean, conversely to estimates from the samples only, which might be driven by sampling and associated environmental biases.

## RESULTS

### C_4_ CCM enzymes across sampled stations

**Fig. 2.**
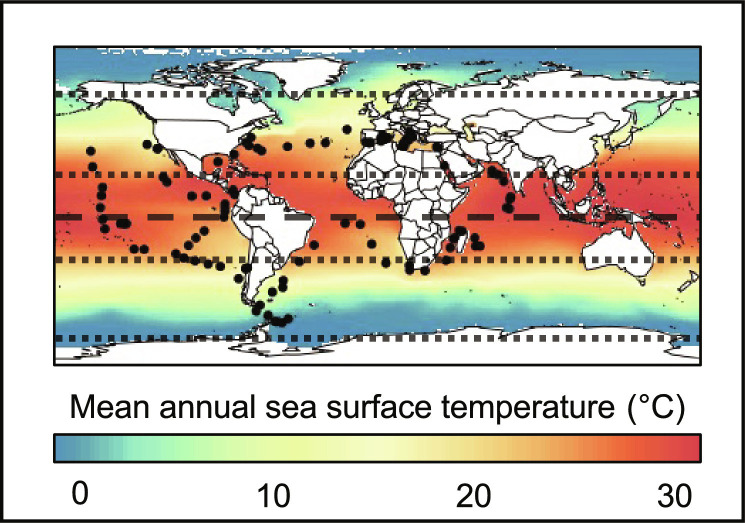
Location of the *Tara* Oceans sampling stations. Stations are represented as black dots. The annual mean sea surface temperature from the World Ocean Atlas (*56*) is represented in the background. The dashed line corresponds to the equator. The dotted lines correspond to the 30°N and 60°N and 30°S and 60°S parallel, respectively.

From the *Tara* Oceans eukaryotic MAGs, ~1.2 million clusters were built, for which 349 are related to RUBISCO or C_4_ enzymes within the 0.8- to 5-μm size fraction (fig. S1 and table S1). This dataset corresponds to 817 unique genes, with a median observed presence across 45 sampled stations per cluster. To avoid considering enzymes related to other metabolic functions, we selected those related to RUBISCO or C_4_ enzymes only, corresponding to 240 clusters (fig. S1A), distributed across the world ocean; except the western Pacific and, to a lesser extent, Southern Ocean (Fig. 2). The successive cluster selection criteria (i.e., clusters exclusive to RUBISCO or C_4_ enzymes, minimum presence at 10 sampling stations) did not present notable effects on the distribution of clusters across number of reads, number of genes, and taxonomic classes (fig. S2). In contrast, we observed a loss of signal for the MDCs (-NAD and -NADP) between functionally exclusive and nonexclusive clusters, highlighting an important fraction of sequence homologs for these enzymes (fig. S2).

### Standardized distribution of the genomic potential related to C_4_ photosynthesis

Here, we present projections for each C_4_ enzyme and the RUBISCO. First, we rescaled the cluster-level projections (i.e., model outputs; fig. S1D) between 0 and 1 (i.e., distribution patterns; fig. S3). Then, we aggregated these patterns at the enzyme level according to their respective functional annotation. We therefore alleviated the propagation of the observed dominance of a given cluster to the aggregated enzyme-level patterns. The resulting enzyme-level projections are referred to as standardized patterns. For each enzyme, it represents a prediction of the genomic potential according to the environmental conditions at each geographical location and independently of any taxonomic dominance.

**Fig. 3.**
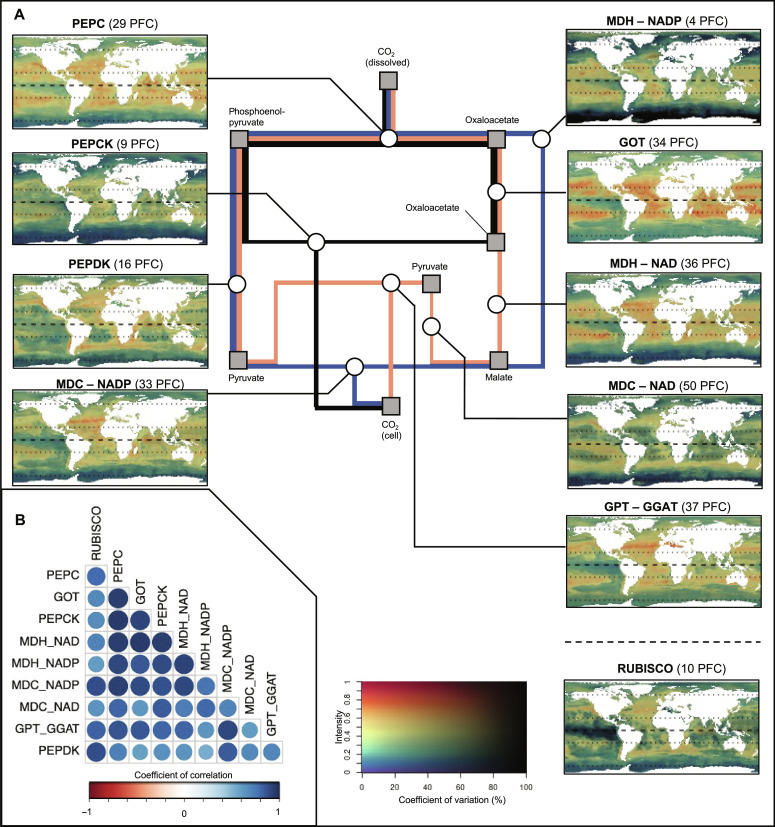
Standardized distributions of the genomic potential. Standardized patterns corresponding to the relative genomic potential supporting C_4_ enzymes and RUBISCO. (**A**) Synthetic diagram of the metabolic pathway and corresponding projections. (**B**) Inter-projections Pearson’s spatial correlation index. The three main currently described acid decarboxylation types are represented in blue (MDC-NADP), orange (MDC-NAD), and black (PEPCK), respectively. Involved metabolic components and enzymes are indicated on the diagram by squares and circles, respectively. The two-dimensional (2D) color scale represents the standardized genomic potential for the target enzyme as the hue value (*y* axis) and the associated coefficient of variation as the saturation (i.e., uncertainty in % of the mean; *x* axis). An orange-to-red hue corresponds to a region where environmental conditions yield a high proportion (>0.6) of the target genes in the model. A low saturation level corresponds to an important variance among the underlying cluster-level projections. The dashed line on the projections corresponds to the equator. The dotted lines on the projections correspond to the 30°N and 60°N and 30°S and 60°S parallel, respectively.

Because most C_4_ enzymes are involved in several acid decarboxylation types, we cannot directly infer their corresponding distribution. However, MDC-NAD, MDC-NADP, and PEPCK are considered representative of their respective acid decarboxylation types. We predicted similar standardized patterns (Fig. 3) for all acid decarboxylation types and RUBISCO. The standardized patterns of all C_4_ enzymes presented medium to high pairwise Pearson’s correlation (0.5 to 0.9), except MDC-NAD and aspartate aminotransferase(s) (also called glutamic-oxaloacetic transaminase, GOT; Fig. 1) which are weakly correlated (0.3). We predicted a medium-to-high genomic potential (between 0.6 and 0.8) for most C_4_ enzymes in temperate and tropical oligotrophic conditions (between 50°N and 40°S, excluding major upwelling areas; Fig. 3). The abovementioned predictions are associated with a coefficient of variation (CV) below 30% (Fig. 3A). The genomic potential of both PEPDK and MDC-NAD, however, presents lower values (between 0.3 and 0.4) in tropical oligotrophic gyres and in the pacific equatorial upwelling for PEPDK. Furthermore, we predicted areas of high genomic potential (>0.8) restricted to temperate areas such as the North and South Atlantic (~50°N and 40°S) and the North Pacific (~45°N) for RUBISCO and PEPCK, in comparison with other C_4_ enzymes. These patterns suggest a higher affinity of the genomic potential of C_4_ enzymes for the temperate and tropical oligotrophic conditions in comparison to RUBISCO. Furthermore, we predicted low-to-moderate potential (between 0 and 0.4) in high latitudes (i.e., above polar circles) for all standardized patterns (Fig. 3A). Predictions in such latitudes also present important calibration and projection-related variability, with coefficients of variations ranging from 30 to 100% (e.g., for the MDH-NADP and PEPCK). Therefore, our genomic potential predictions remain inconclusive in high latitudes, which are also subject to lower sampling coverage.

The environmental variables’ importance in the trained model (fig. S4) highlighted the predominant roles of dissolved oxygen concentration (contributing to 34% of the explained variance) and the yearly variability (i.e., inter-month SD) in salinity (29%) and, to a lesser extent, of oxygen saturation, chlorophyll a concentration, and temperature. Furthermore, we revealed a strong affinity (i.e., maximum potential) of most standardized patterns (fig. S5) for tropical, oligotrophic conditions (e.g., temperature between 15° and 30°C; phosphate concentration below 0.5 μmol/kg). However, we predicted different responses to the variability in chlorophyll a concentration and euphotic zone depth across enzymes (fig. S5). Last, we highlighted no taxonomic dominance across the world oceans, according to the taxonomic composition associated with each cluster, suggesting a worldwide functional redundancy in the genomic potential supporting C_4_ enzymes in piconanoplankton (fig. S6).

### Weighted distribution of the genomic potential related to C_4_ photosynthesis

Here, we present projections for each C_4_ enzyme and the RUBISCO. First, we rescaled the cluster-level projections (i.e., model outputs; fig. S1D) by their observed metagenomic read abundance (i.e., weighted distribution patterns; fig. S3). Then, we aggregated these patterns at the enzyme level according to their respective functional annotation. We therefore propagate the observed dominance of a given cluster (i.e., and associated taxa) to the aggregated enzyme-level patterns. The resulting enzyme-level projections are referred to as weighted patterns. For each enzyme, it represents the corresponding genomic potential (i.e., relative to the other considered enzymes), according to the environmental conditions at each geographical location.

**Fig. 4.**
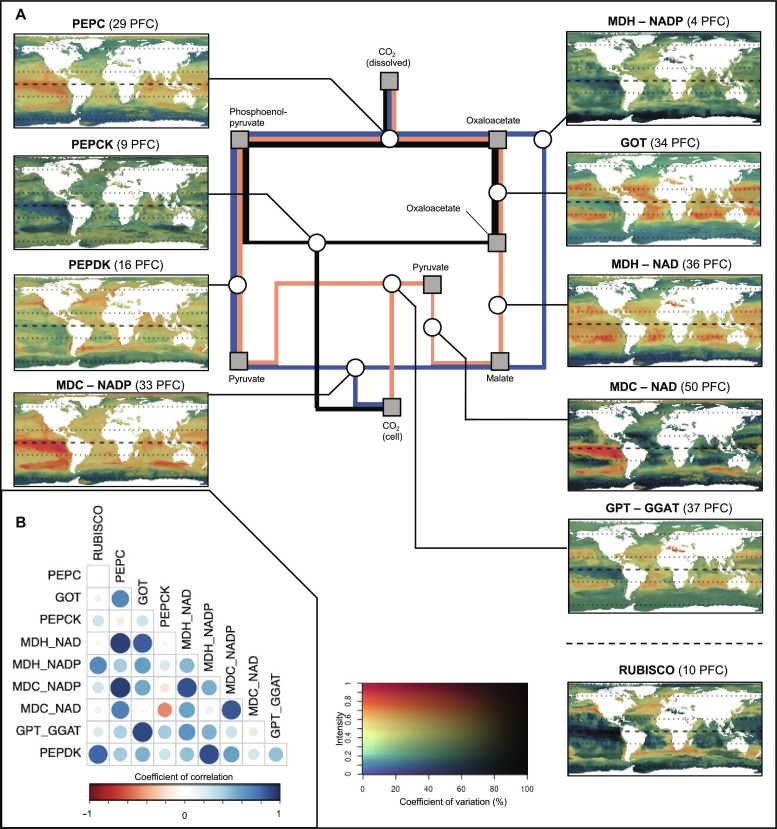
Weighted distributions of the genomic potential. Weighted patterns corresponding to the relative genomic potential supporting C_4_ enzymes and RUBISCO, rescaled by the corresponding observed relative metagenomic reads abundance. (**A**) Synthetic diagram of the metabolic pathway and corresponding projections. (**B**) Inter-projections Pearson’s spatial correlation index. The three main currently described acid decarboxylation types are represented in blue (MDC-NADP), orange (MDC-NAD), and black (PEPCK), respectively. Involved metabolic components and enzymes are indicated on the diagram by squares and circles, respectively. The 2D color scale represents the weighted genomic potential for the target enzyme as the hue value (*y* axis) and the associated coefficient of variation as the saturation (i.e., uncertainty in % of the mean; *x* axis). An orange-to-red hue corresponds to a region where environmental conditions yield a high proportion (>0.6) of the target genes in the model. A low saturation level corresponds to an important variance among the underlying cluster-level projections. The dashed line on the projections corresponds to the equator. The dotted lines on the projections correspond to the 30°N and 60°N and 30°S and 60°S parallel, respectively.

We predicted contrasting weighted patterns between the RUBISCO and the acid decarboxylation types (Fig. 4A). The weighted pattern of RUBISCO presented maximum potential in temperate areas (Fig. 4B). We predicted low-to-moderate potential (<0.3) and moderate (~30%) uncertainty in high latitudes for the weighted patterns of PEPC, MDCs, MDHs, and transferases (i.e., GOT and alanine transaminase; GPT-GGAT; Fig. 4A). These patterns also presented moderate-to-high potential (between 0.5 and 1) in tropical areas, with some discrepancies. We show a Pearson’s correlation index above 0.5 between the abovementioned enzymes and above 0.7 for GOT and MDHs (Fig. 4B). The latter presented an important potential in oligotrophic regions (e.g., Pacific gyres), suggesting functional redundancy in the genomic potential from oxaloacetate to malate (Fig. 4A). In contrast, we predicted a high potential (>0.7) in eutrophic Pacific waters for the weighted patterns of MDCs (Pearson’s correlation above 0.7; Fig. 4A). Overall, we show high confidence in the areas associated with high genomic potential, with CVs lower than 30% among all trained algorithms and 100 bootstrap projections. The abovementioned weighted responses to environmental variables are like the ones highlighted in the previous section, characterized by higher potential in warm, low seasonality, and generally oligotrophic water bodies (figs. S7 and S8).

Conversely, we predicted moderate to high-intensity values in oligotrophic tropical areas, but in the Southern Ocean (>0.5; Fig. 4) for the weighted pattern of PEPCK (i.e., a different acid decarboxylation type). The latter was preferentially distributed along water bodies characterized by (i) high seasonality of the chlorophyll a concentration and the depth of the euphotic zone, (ii) high concentrations of oxygen (presenting the highest explanatory power in the model training; fig. S4) and nutrients (e.g., phosphates and nitrates), and (iii) average temperatures below 8°C (fig. S7).

Last, weighted patterns associated with high latitudes (e.g., correlated with the one of PEPCK) were composed at 28% of Prymnesiophyceae and 50% of Mamiellophyceae (Shannon index of 1.5), based on the taxonomic composition of each cluster. Mamiellophyceae also composed 40% of the patterns with a clear temperate affinity (e.g., correlated with the one of RUBISCO; fig. S8). In contrast, a larger diversity of taxonomic classes, with a Shannon index of 2.1, was obtained for patterns associated with equatorial latitudes.

## DISCUSSION

### Genomic potential for C_4_ CCM in piconanoplankton

By selecting clusters annotated by C_4_ enzymes or RUBISCO only, we considered a fraction of the available metagenomic information (i.e., ~67% of all the clusters related to C_4_ enzymes or RUBISCO). In addition, genes related to other metabolic pathways may have responses to environmental variables different from genes related to C_4_ enzymes, potentially including bias in their corresponding clusters’ projection. Therefore, selecting a reduced set of clusters alleviates the risk of metabolic noise in the environmental responses, limited to the effect of C_4_ enzymes potentially involved in other pathways (e.g., GPT-GGAT transporter).

Our study focused on piconanoplankton, the photosynthetic fraction of which is generally dominated by the Prymnesiophyceae, Bacillariophyceae, Dinophyceae, and Mamiellophyceae lineages in the open ocean (*3*, *21*). The latter is a major clade of the polyphyletic Prasinophyceae assemblage (*37*). The potential for C_4_ photosynthesis has been suggested for several families, including Bacillariophyceae by combining C_4_ enzyme inhibition and photosynthetic efficiency monitoring [e.g., PEPDK (*38*), PEPC, and PEPCK (*39*)]. Evidence for genes encoding all C_4_ enzymes exists in *Micromonas* and *Ostreococcus*, both belonging to the Mamiellophyceae (*37*, *40*). A plastid PEPC enzyme was recently found in *E. huxleyi* (*38*), a Prymnesiophyte abundant in temperate and polar regions (*41*). However, to our knowledge, no study provided univocal evidence for C_4_ CCM usage in natural populations for the smallest fraction of piconanoplankton, contrasting with recent findings supporting C_4_ CCM usage by marine diatoms (*14*). Stable isotope measurements would be necessary to fully understand C_4_ photosynthesis in piconanoplankton, but they are difficult to apply at the species level in natural, uncultured, plankton communities [see, e.g., (*16*, *17*)]. Alternatively, recent literature suggests the need for further studies on deep chlorophyll a maxima and various transporters (e.g., bicarbonate transporters), some of which are associated with or specific to C_4_ metabolism, to better understand C_4_ CCM in natural populations (*14*, *15*).

Complementing these experimental approaches, we use a data-driven approach to shed more light on the environmental drivers of C_4_ genes in marine piconanoplankton. However, MAGs integrate chloroplast and mitochondrial genes corresponding to C_4_ enzymes but do not distinguish their origin (*29*) nor provide information on the subcellular location of the corresponding enzymes (*13*, *42*). Therefore, the patterns presented here must be interpreted as the potential for the (co-)presence of those pathways in the genome. They should now be complemented by culture-based studies, locating enzymes within cells and/or performing carbon isotope discrimination to confirm C_4_ CCM presence, expression, and its coexistence with C_3_ photosynthesis in piconanoplankton lineages (*16*). The present study could be used to locate regions where such mechanisms are most likely to occur.

### Environment-driven genomic potential

The modeled distribution patterns revealed that the genomic potential for C_4_ photosynthesis is more associated with tropical oligotrophic and annually stratified waters. Conversely, the proportion of reads related to RUBISCO (i.e., considered a representative of all photosynthetic pathways, due to its central role in C_3_, C_4_, and CAM photosynthesis) is higher in temperate regions (Fig. 3A). The fact that terrestrial C_4_ plants (*8*) and the genomic potential for C_4_ CCM in piconanoplankton display similar latitudinal distribution, around the tropics, does not imply that the environmental drivers of those distributions are the same. In terrestrial plants, C_4_ CCMs are considered an adaptation to drought and are, for example, also associated with a specific leaf structure that reduces their water consumption (*8*). Drought is of course not an evolutionary driver for marine piconanoplankton. Alternatively, they present an important surface area:volume ratio [i.e., small cells or presence of a vacuole (*43*, *44*)] leading to a high nutrient absorption yield, which is adapted to oligotrophic waters, common in the tropical ocean.

In addition to environmental conditions, the biogeography of the genomic potential supporting C_4_ CCM may also relate to irradiance levels, largely controlling ATP generation, necessary for the decarboxylation reaction (*43*). C_4_ CCM requires additional ATP generation to increase the RUBISCO efficiency in comparison to classical C_3_ photosynthesis without affecting the energy available for the latter (*43*, *45*). In contrast, an excess of ATP may lead to photoinhibition, thus lower carbon fixation efficiency (*38*, *46*). Therefore, it has been suggested that C_4_ photosynthesis is particularly adapted to dissipate excess energy in the cell in high irradiance areas such as tropical oceans (*14*, *38*). Our weighted patterns highlighted differences between PEPCK and MDCs (Fig. 4). The latter requires two extra ATPs compared to the C_3_ carbon fixation to complete the pathway. In a logical way, the PEPCK acid decarboxylation type, which only requires one extra ATP and thus is supposed to be more efficient in low irradiance environments (*45*), showed here the highest genomic potential in polar or subpolar regions.

### Functional and ecological implications

We highlighted functional redundancy among C_4_ genes in oligotrophic tropical waters (fig. S6). This contrasts with high latitudes, where only a few taxa dominate (fig. S8) (*4*, *47*). We highlighted a biogeographical differentiation between the weighted pattern of RUBISCO—i.e., the baseline photosynthetic enzyme—and those of C_4_ enzymes. In the period ranging from 30 million to 20,000 years ago, the average atmospheric CO_2_ concentration markedly reduced from ~1000 ppm to less than 200 ppm. This long-term atmospheric CO_2_ concentration trend induced a lower concentration of dissolved inorganic carbon in the surface ocean waters (*8*). This led to a selective pressure toward efficient photosynthetic metabolism, like C_4_ CCMs (*12*) or, to a lesser extent, RUBISCO of higher carboxylation affinity [e.g., type II in Dinoflagellates (*13*)]. While the evolution of C_4_ CCMs in marine organisms is not yet fully understood, 48 independent evolutions of C_4_ CCMs were identified in the genome of terrestrial plants [e.g., grasses and caryophyllales (*8*)], suggesting a higher genomic potential for C_4_ CCMs in taxonomically diverse areas (*12*). The abovementioned functional redundancy in the genomic potential for C_4_ CCM in taxonomically rich tropical waters may relate to coevolution between taxonomic diversification and its associated functions (i.e., neutral theory). However, the functional diversity among C_4_ acid decarboxylation types may also reflect—or be amplified by—a selection process, as it may present a selective advantage. Moreover, the dominance of Mamiellophyceae (i.e., relative to other piconanoplankton associated with RUBISCO or C_4_ enzymes) in the temperate and polar latitudes (associated with the patterns of RUBISCO and PEPCK; fig. S8) is concordant with the literature (*48*). Although cosmopolitan, several Mamiellophyceae species have been shown as important in both the Arctic and Antarctic [see, e.g., (*49*–*51*)]. Likewise, the cosmopolitan Prymnesiophyceae has been identified as dominant in high latitudes (associated with the pattern of PEPCK; fig. S8), including in the Southern Ocean (*41*) and associated with Mamiellophyceae (i.e., Prasinophyceae) in the North Atlantic (*52*, *53*). The literature therefore validates their predicted biogeography. We identified key environmental predictors shaping the biogeography and (co-)dominance patterns of the genomic potential supporting C_4_ enzymes and RUBISCO in marine piconanoplankton. Such results open perspectives for exploring the relationship between functional and taxonomic diversity in the oceans, complementing already diverse approaches and data types, and for a better understanding of the environmental drivers of key biogeochemical cycles in the current and future climatic context.

## MATERIALS AND METHODS

### Experimental design

#### Genomic and environmental data

We studied the biogeography of the genomic potential related to C_4_ enzymes through the prism of MAGs (*29*) retrieved from the *Tara* Oceans expedition (2009–2013). Briefly, 280 billion reads from 798 metagenomes, corresponding to the surface and deep chlorophyll maximum layer of 210 stations from the Pacific, Atlantic, Indian, Southern, and Arctic Oceans, as well as the Mediterranean and Red Seas (Fig. 2), encompassing eukaryote-enriched plankton size fractions ranging from 0.8 μm to 2 mm, were used as inputs for 11 metagenomic coassemblies (6 to 38 billion reads per coassembly) using geographically bounded samples. We thus created a culture-independent, nonredundant (average nucleotide identity <98%) genomic database for eukaryotic plankton in the sunlit ocean consisting of 683 MAGs and 30 single-cell genomes, all containing more than 10 million nucleotides for a total size of 25.2 Gbp and encoding for 10,207,450 genes. Then, a sequence similarity network (SSN) was built out using the 683 manually curated MAGs following a similar methodology to the one developed in (*33*). A pairwise comparison was computed between each protein sequence. The resulting alignment was then filtered, removing self-hits and pairs showing less than 80% of sequence identity and coverage. Resulting PFCs [as in (*33*)] were built, hereafter referred to as clusters. A functional annotation was added to the sequences, and the functional homogeneity was checked in each cluster (*54*, *55*).

For each of the 130 selected *Tara* Oceans metagenomic surface samples, we retrieved a set of monthly, global scale, environmental climatologies (*56*–*58*) encompassing the 2005 to 2017 period, at a spatial resolution of 1° × 1° (table S2). The latter corresponds to the available climatology encompassing the sampling period (2009–2013), where we considered temporal environmental variations negligible in comparison to spatial environmental gradients. They correspond to a restricted set of factors characterizing the water body (e.g., oligotrophic and eutrophic) and related to C_4_ photosynthesis, for which we calculated the yearly average and yearly SD (i.e., a proxy of seasonal variations).

#### Protein functional cluster selection and preprocessing

We first selected a reduced set of clusters, within the 0.8- to 5-μm size fraction, for which 100% of the Kyoto Encyclopedia of Genes and Genomes orthology (*59*) annotated protein members were related to C_4_ enzymes or RUBISCO (fig. S1 and table S1). To avoid model overparameterization and because rare clusters were assumed as not influencing the large-scale patterns investigated in this study, we only considered clusters that were present in a minimum of 10 *Tara* Oceans stations. The corresponding dataset contained 240 clusters, associated with 234 MAGs. The latter presented an average completeness estimate of 57% (data S1). In comparison, the average completeness estimate across all MAGs from Delmont *et al*. (*29*) yields 37%. As a supplementary quality check, we estimated a minimum horizontal coverage (i.e., the number of bases of a MAG covered with a certain depth) of 68% for each of the 234 MAGs (data S1). Last, we assessed the quality of our MAGs using the Benchmarking Universal Single-Copy Orthologs (BUSCO) protocol (*60*). The latter is a set of conserved single-copy genes present in most eukaryotic and prokaryotic genomes. It is used to assess both the completeness and quality of genomic data by comparing the presence of conserved single-copy genes across genomes (i.e., the percentage of mapped BUSCO genes in each MAG). It therefore complements technical metrics such as contiguity measures and is largely applicable across datasets (*60*). We show that our MAGs are associated with an average BUSCO completeness of 55.7% (data S1). We therefore consider these MAGs of sufficient quality for identifying C_4_ genes across our samples. To reduce the number of response variables (clusters; PFCs) to a reasonable amount for multivariate modeling, with respect to the limited number of stations, we performed an Escoufier dimensional reduction (*61*). The latter iteratively selects the clusters whose pattern across stations minimizes the residual variance of the dataset. Here, we selected 50 clusters that represent more than 95% of the 240 clusters variance to be included in the multivariate algorithm. To alleviate the effect of gene length and sequencing effort variability between samples on the number of reads, we normalized the metagenomic reads by the length of the corresponding gene coding part and the total number of reads per station (i.e., including reads of all non-considered clusters), respectively. Because the total genomic material present at each sampling station is unknown (i.e., non-exhaustive sampling and sequencing effort), the absolute number of reads is not comparable among stations. To compare the abundance between selected clusters at different sampling stations, we transformed the dataset to relative abundance (fig. S1).

#### Multivariate boosted tree regressors

##### General principle

Recently, growing interest in interactions between response variables led to the development of multivariate machine learning algorithms, such as MBTRs (*36*). The latter is particularly adapted to a small sample size as the interactions between response variables are considered supplementary information to calibrate the model. Here, we use MBTR to model the relationship between climatologies and metagenomic relative abundance (i.e., summed at 1 for each station; fig. S1). To best reproduce the response of metagenomic reads (i.e., response variable) to the corresponding environmental variables (i.e., explanatory variable), the model sequentially fits decision trees (i.e., boosting rounds) using gradient descent to minimize a specific loss function. At each boosting round, the algorithm fits a decision tree on the residuals of the previous boosting round and computes a tree loss (i.e., a measure of deviation between observed and predicted response variable values). Decision trees are constructed using the hessian of the loss function (i.e., second-order tensor of its partial derivatives) to minimize the loss gradient. Therefore, the information learned by the *n*th tree is passed to the *n*+1th tree at a user-defined learning rate (fig. S1). The ensemble of sequentially fitted decision trees is considered in the model until the minimum loss is reached. Last, one important feature of MBTR is the conservation of the initial correlation structure between the response variables [see methods in (*36*)].

##### Model training and evaluation

To avoid overfitting, the explanatory and response datasets were split between the training set and the test set using a *n*-fold cross-validation procedure. For each model, *n* algorithms were trained on different *n*−1-folds, while the remaining fold was used for testing only (i.e., computing the loss at each boosting round). To minimize the effect of spatial and temporal autocorrelation in our data [i.e., leading to overoptimistic model evaluation (*62*)] the *n*-folds were defined according to the *Tara* Oceans station number. The latter follows a continuous trajectory in space and time, resulting in spatially and temporally distant folds [i.e., spatial and temporal block splitting, as recommended in (*62*)]. The resulting *n* algorithms predictions were aggregated in an average response and its corresponding CV. The ability of the final model to reproduce the observed clusters’ relative abundance across environmental conditions has been measured by the *R*^2^ criteria and the root mean square error (RMSE; between 0 and 1 according to the distribution pattern scale).

##### Spatial projections

To better estimate projection uncertainty, our spatial projections were constructed using a bootstrap procedure. For each 100-bootstrap round, we first resampled the original dataset (i.e., train and test response dataset and corresponding explanatory variable values) with replacement. Then, we refitted an MBTR algorithm on the resampled data by using the hyperparameters corresponding to the validated model, including the number of boosting rounds corresponding to the minimum loss across all *n* algorithms. Last, the refitted MBTR algorithm was used to predict the relative abundance of clusters worldwide, using the corresponding climatological values at each geographical cell.

#### From model projections to final outputs

We only modeled the 50 clusters representing 95% of the dataset variability. Therefore, we indirectly reconstructed the projections of the 190 others by identifying their most representative Escoufier-selected cluster. To this extent, we performed a correspondence analysis based on the observed relative abundance of all clusters. By using the dimensions of the correspondence analysis space corresponding to a minimum of 80% variance explained, we calculated the Euclidean distance between each nonselected cluster, and its nearest neighbor selected by the Escoufier criteria. Because the 50 Escoufier-selected clusters represented more than 95% of the dataset variability, we considered that a cluster and its nearest neighbor in the correspondence analysis space share the same relative abundance pattern. We then reconstructed the spatial projections of the 190 clusters not considered in MBTR according to their projected nearest Escoufier-selected neighbor. The resulting 240 cluster-level projections of the genomic potential were then aggregated at the enzyme level according to their functional annotation (see Results; fig. S3). Each projection was standardized between 0 and 1, thus considered equally weighted distribution patterns (fig. S3, top). We then performed two aggregation methods, leading to a (i) standardized and a (ii) weighted distribution of the genomic potential related to C_4_ photosynthesis. In the former, we performed a simple average of all cluster-level projections sharing a similar functional annotation (fig. S3, left). This resulted in an enzyme-level projection reflecting the most common patterns at the cluster level. In other words, the highest genomic potential at the enzyme level was located where most cluster-level projections present their highest genomic potential (fig. S3, left). It was independent of any taxonomic dominance. In the latter, however, we performed a weighted average of all cluster-level projections sharing a similar functional annotation. The weights corresponded to the sum of the observed relative abundance of each cluster, across all stations (figs. S1A and S3, right). This resulted in an enzyme-level projection reflecting the cluster-level patterns with the highest relative abundance (i.e., dominant patterns). In other words, the highest genomic potential at the enzyme level was located where abundant cluster-level projections presented their highest genomic potential (fig. S3, left). It propagated the associated taxonomic dominance to the enzyme-level patterns.

### Statistical analyses

#### Metagenomic data construction

The bioinformatic workflow designed to build the MAGs can be found in (*63*) and on the genoscope website: www.genoscope.cns.fr/tara/. Original metagenomes are available under the European Bioinformatics Institute repository with project ID PRJEB402 and organized into four major size classes of 0.8 to 5 μm, 3 to 20 μm, 20 to 180 μm, and 180 to 2000 μm. To estimate the abundance and expression of each contig in each sample, cleaned reads (from metagenomes and metatranscriptomes) were mapped against the eukaryotic MAGs using the bwa tool (version 0.7.4). The following parameters were used: bwa aln -l 30 -O 11 -R 1; bwa sample -a 20000 -n 1 –N; samtools; rmdup. Reads covering at least 80% of read length with at least 95% of identity were retained for further analysis. In the case of several possible best matches, a random one was picked. The first SSN was built out of 683 manually curated MAGs from 10,207,435 eukaryotic proteins. This file was used for the creation of a diamond database and a protein blast of the protein sequences against the database to compute the percentage of similarity between every pair of proteins detected in the MAGs. Here, we used a maximum *e*-value of 1 × 10^−3^ and the sensitive option adapted to long reads (−e 1e^−3^ −p 30–sensitive). The alignment was then filtered removing all self-hits. Several thresholds for the percentage of identity and coverage were tested (75, 80, 85, and 90%). An SSN (bioinformatic workflow available at https://data.d4science.net/BN9t) was built with the diamond output using 80% identity and 80% coverage threshold to minimize the number of singletons while maximizing the functional homogeneity between linked proteins. Reproducible analysis and statistical exploration are provided in (*33*, *54*, *64*). An SSN is made of singletons (vertices or sequences without any homology with other sequences) and connected components (CCs; i.e., subgraphs composed of at least two vertices disconnected from the rest of the network). In our case, a CC corresponds to a group of at least two protein sequences that are linked together (directly or via neighbors) and that have no link with other groups of sequences in the SSN. We assume that the proteins contained in a CC potentially share a similar molecular function (*54*, *55*, *64*, *65*). These proteins were functionally annotated using eggNOG mapper v2.1.5.

#### Data selection

Unless specified, all following analyses were performed using R 3.14, with the corresponding code and libraries available at https://data.d4science.net/qa7Z or 10.5281/zenodo.11093527. Starting from station no. 66 (i.e., Cape Town), a supplementary size class of 0.8 to 2000 μm has been implemented in the *Tara* Oceans cruise, while the initial 0.8 to 5 μm was not sampled from stations 155 onward (i.e., Arctic stations). Given that smaller organisms are much more abundant than large ones, the majority of organisms sampled with the 0.8- to 2000-μm filter are piconanoplankton (i.e., corresponding to 0.8 to 5 μm). We tested this hypothesis by analyzing the composition, detection rate, and associated percentage of metagenomic reads across clusters associated with RUBISCO or C_4_ enzymes, between 0.8 to 5 μm and 0.8 to 2000 μm at their common sampling stations (i.e., 66 to 155). The composition of clusters of interest across common stations presented a significant correlation (Pearson correlation: 0.89; *P* value: 0.01) between both size fractions. Moreover, 85.5% of the abovementioned clusters detected in the 0.8- to 2000-μm fraction were also detected in the 0.8- to 5-μm fraction. The latter represents 86.3% of the mapped reads in the 0.8- to 2000-μm fraction. The clusters detected in both fractions at common locations present a Pearson and Spearman metagenomic read correlation of 94.4 and 87.3%, respectively. This supports the inclusion of Arctic data issued from the 0.8- to 2000-μm filter. The effect of the different selection criteria such as the exclusivity and the minimum number of stations coverage is shown in fig. S3 and calculated using Pearson’s chi-square test for count data (“chisq.test” function; *P* < 0.05).

#### Model training, evaluation, and projections

We fitted one MBTR (Python >3.7.) algorithm per training set and hyperparameter combination, under a mean square error loss function, a learning rate of 5.10^−3^, and a 10-fold cross-validation procedure. We set the minimum number of observations in terminal leaves to 30 and the number of quantiles considered to find the best split to 10. The model loss is adapted to discriminate between different sets of hyperparameters. Thus, we only considered the set of hyperparameters that resulted in the minimum loss across the corresponding 10 trained MBTR algorithms. However, the loss does not provide information on the actual performance of the model in reproducing the observed data. Therefore, for each of the 10 trained MBTR algorithms, we predicted the relative abundance of the metagenomic reads on the environmental values corresponding to each of the 10 test sets. The 10 corresponding predictions were compared against the truth (i.e., observed values) by means of the *R*-squared (*R*^2^) and RMSE (here between 0 and 1) to assess model performance on data not seen by the MBTR models during the training process. The corresponding model evaluation estimated an *R*^2^ of 0.33 and an RMSE of 0.05. The conservation of the correlation structure in MBTR was tested by computing a Pearson correlation matrix between response variables before and after model fitting, followed by a Mantel test (Pearson’s *R* = 0.748; *P* = 0.01). Last, for the spatial projections, we performed a total of 100 bootstrap rounds and computed the average and CV between all bootstrap projections (fig. S1).

#### Model outputs

The variable importance in the model training (fig. S4) was calculated as the number of times an environmental variable was selected for a tree split, scaled by the corresponding loss gain. The response of the genomic potential for an enzyme to each environmental variable was estimated by partial dependence plots. The latter was defined as the marginal response of the target to a feature over the values of all other input features. To estimate the taxonomic composition associated with each pattern (i.e., standardized, or weighted), we constructed the distribution pattern of each MAG using the same modeling framework. We then performed a hierarchical clustering [the “ward.D2” method (*66*)] on MAGs level projections that were then correlated to the enzyme’s distributional patterns (Pearson’s *R* correlation). Last, according to each MAG annotation, we computed the taxonomic composition corresponding to each cluster of MAG patterns.
