# Supplementary material for: The genomic potential of photosynthesis in piconanoplankton is functionally redundant but taxonomically structured at a global scale
Source: Sci Adv. 2024 Aug 16;10(33):eadl0534. doi: 10.1126/sciadv.adl0534 (PMC11328907; doi:10.1126/sciadv.adl0534)
Supplement: Supplementary file 1 — Figs. S1 to S8 Tables S1 and S2 Legend for data S1 [file sciadv.adl0534_sm.pdf]

Science Advances
AAAS

Supplementary Materials for

The genomic potential of photosynthesis in piconanoplankton is functionally redundant but taxonomically structured at a global scale

Alexandre Schickele *et al.*

Corresponding author: Alexandre Schickele, alexandre.schickele@imev-mer.fr

Sci. Adv. **10**, eadl0534 (2024)
DOI: 10.1126/sciadv.adl0534

The PDF file includes:

Figs. S1 to S8
Tables S1 and S2
Legend for data S1

Other Supplementary Material for this manuscript includes the following:

Data S1

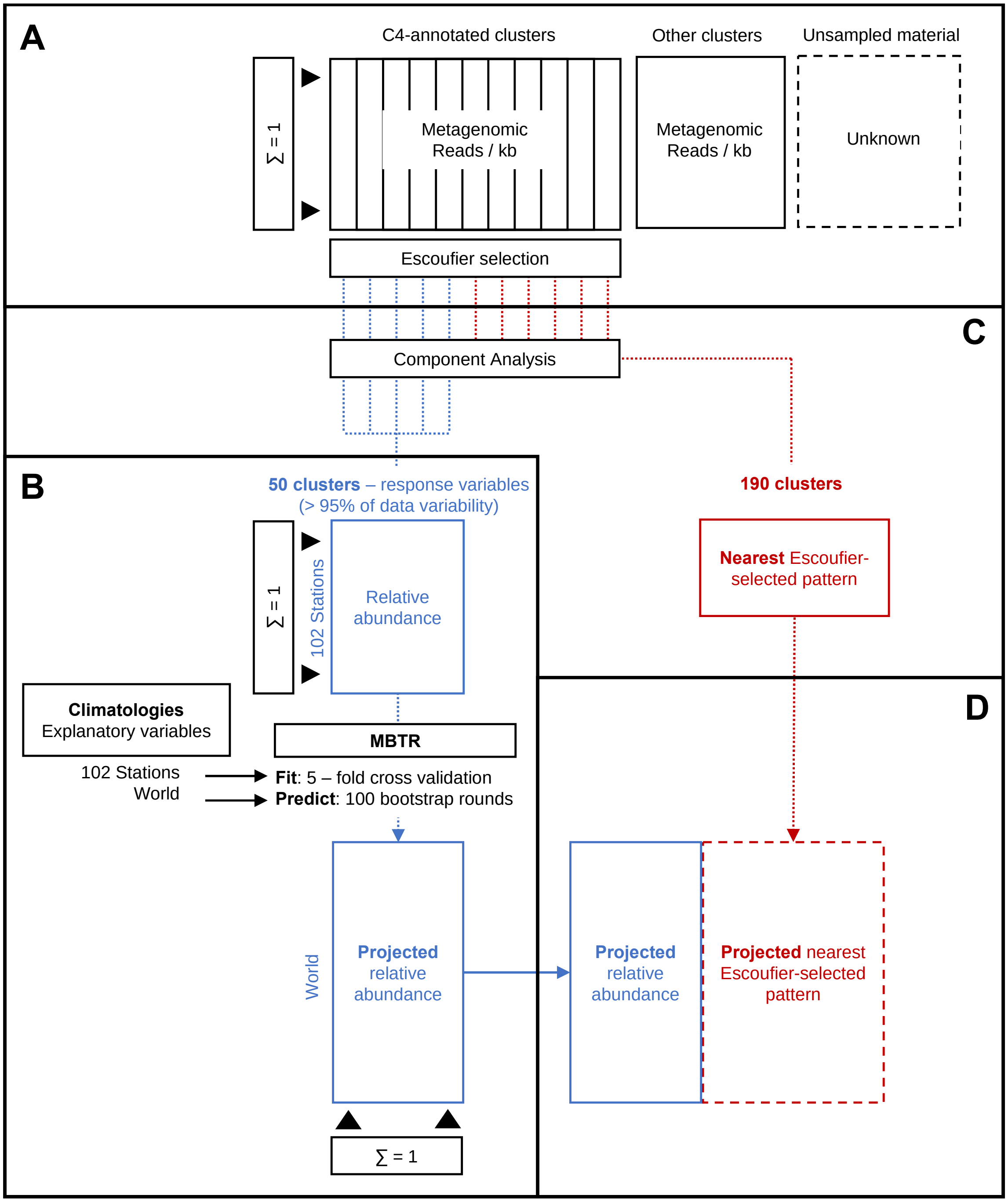

Fig. S1.

Synthetic diagram presenting the successive modelling steps, including (A) the genomic data selection and pre-processing, (B) the Multivariate Boosted Tree Regressor (MBTR) training and projections, (C) the consideration of protein functional clusters not selected in MBTR and (D) the resulting projections at the cluster-level.

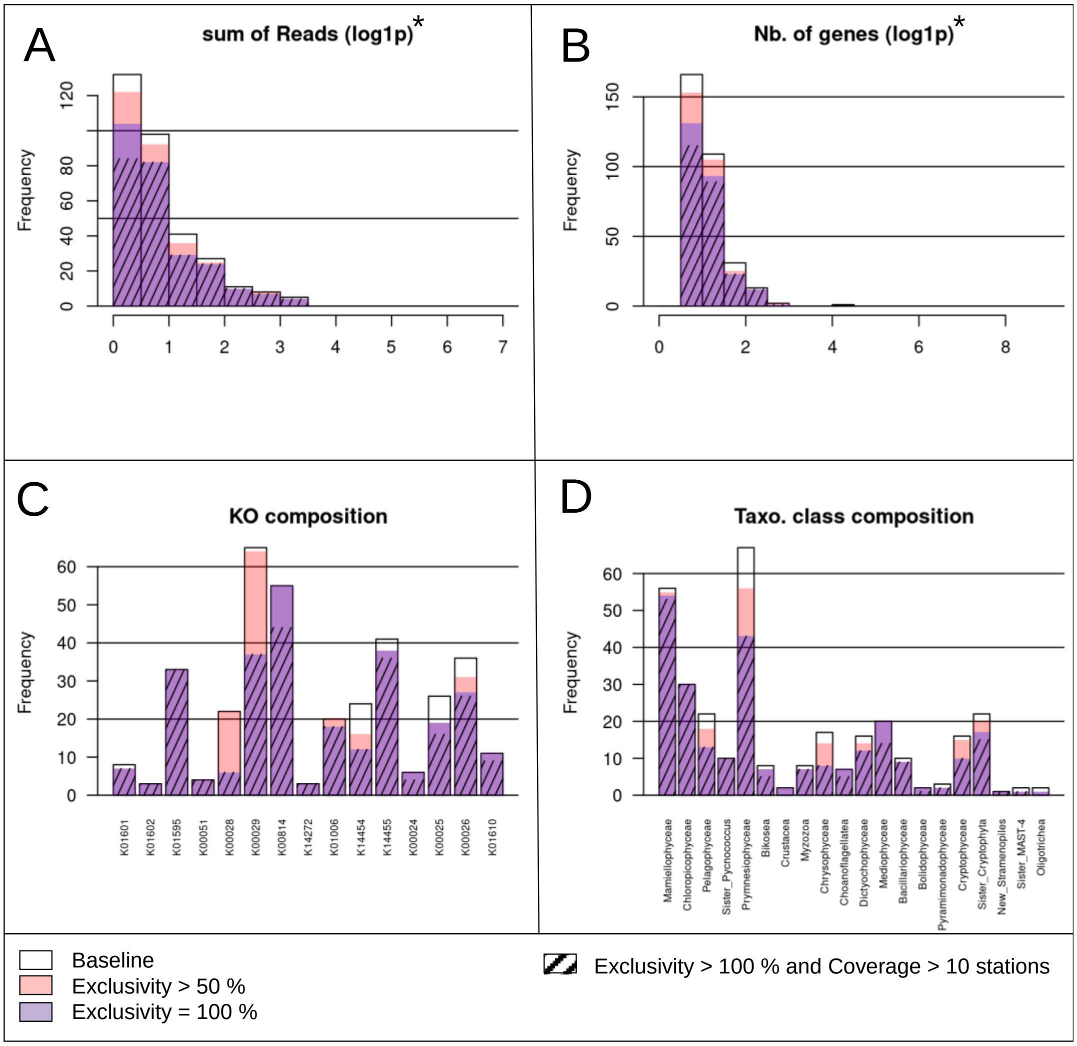

* all filters have significantly the same distribution as "Baseline" with 95% significance

Fig. S2.

Effect of various protein functional cluster selection criteria on their composition.

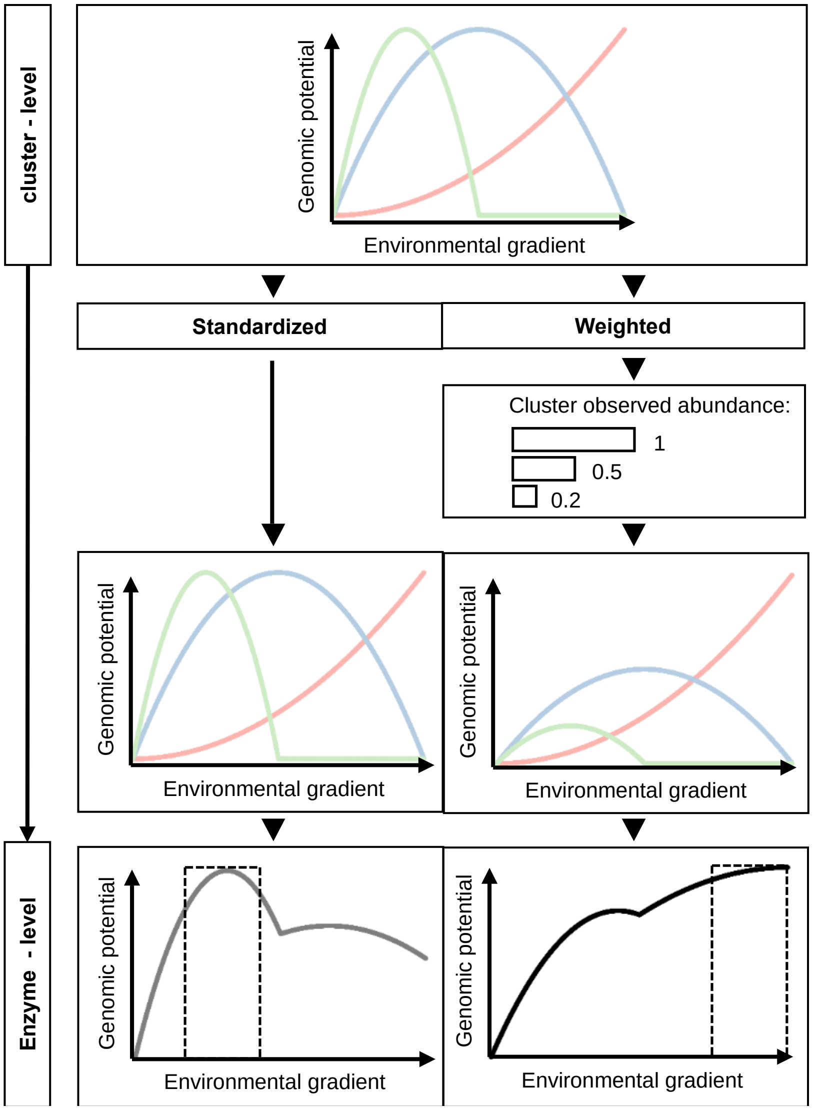

Fig. S3.

Synthetic diagram describing the pattern aggregation methods from the cluster-level to the enzyme-level. The left panels represent the construction of standardized patterns, where all cluster-level patterns are aggregated with equal weight into the enzyme-level pattern. The right panels represent the construction of weighted patterns, where each cluster-level pattern is weighted according to its observed abundance when aggregated into the enzyme-level pattern. The dashed box in the bottom panels represents the highest genomic potential corresponding to each aggregation method, with respect to the environmental gradient.

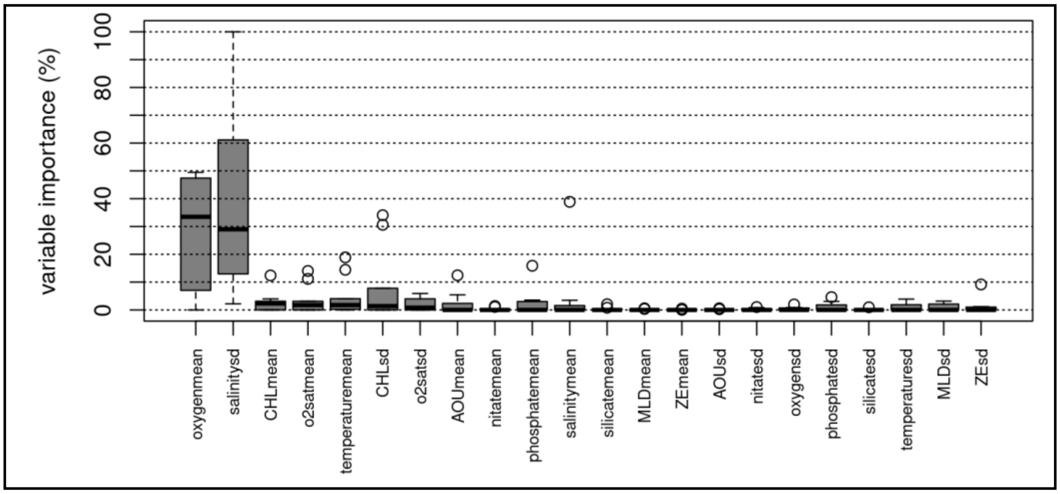

Fig. S4.

Environmental variable importance in model training, across cross-validation runs. The midline of the boxplots corresponds to the median, with the upper and lower box limits corresponding to the first and third quartile. Whiskers extend to 1.5 times the interquartile range and white points correspond to outliers.

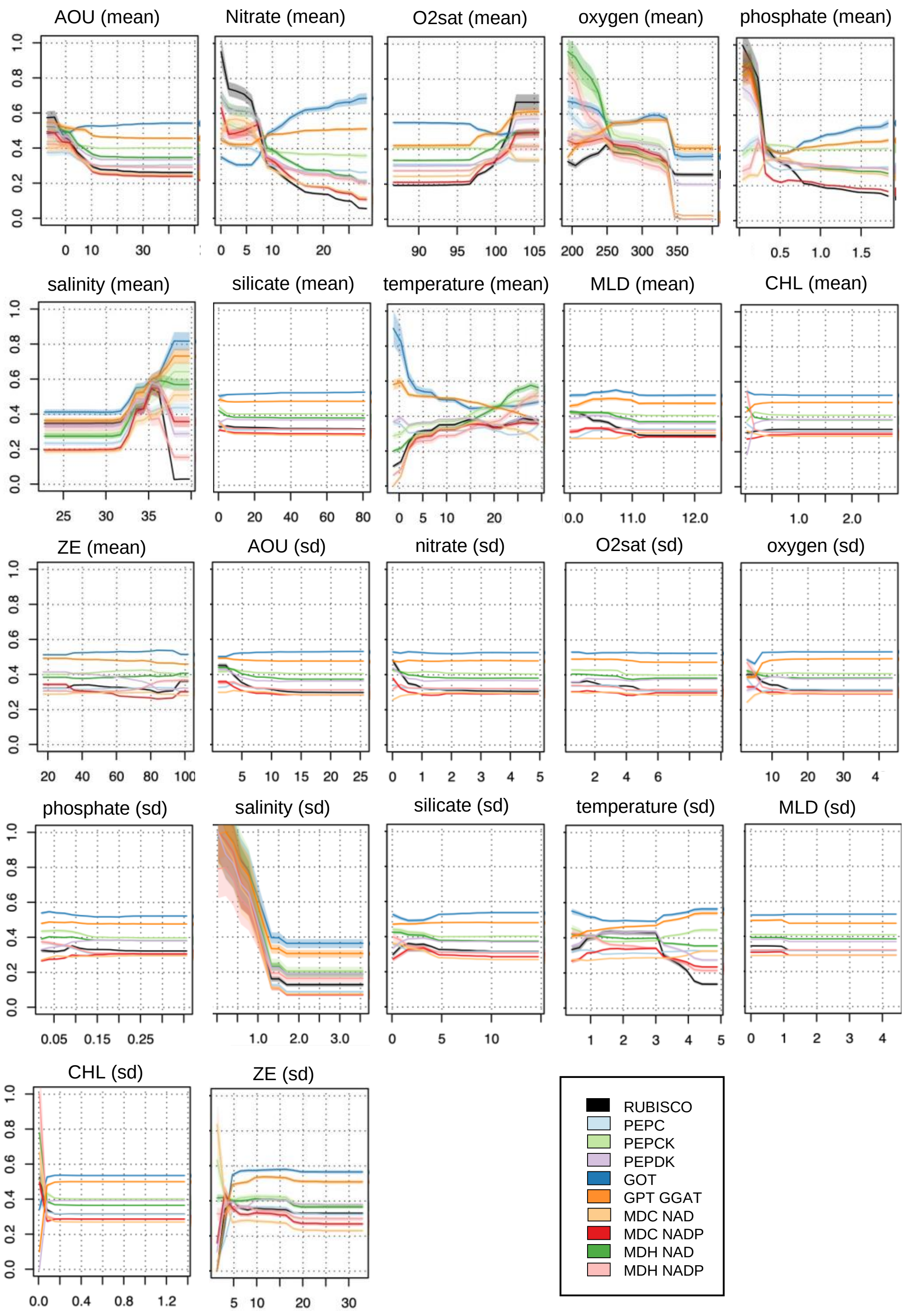

Fig. S5.

Partial dependence plots corresponding to the standardized pattern. The Y axis corresponds to the genomic potential while the X axis represents the environmental parameter possible values across world oceans. The full line and shading respectively correspond to the average response and standard deviation between bootstrap runs.

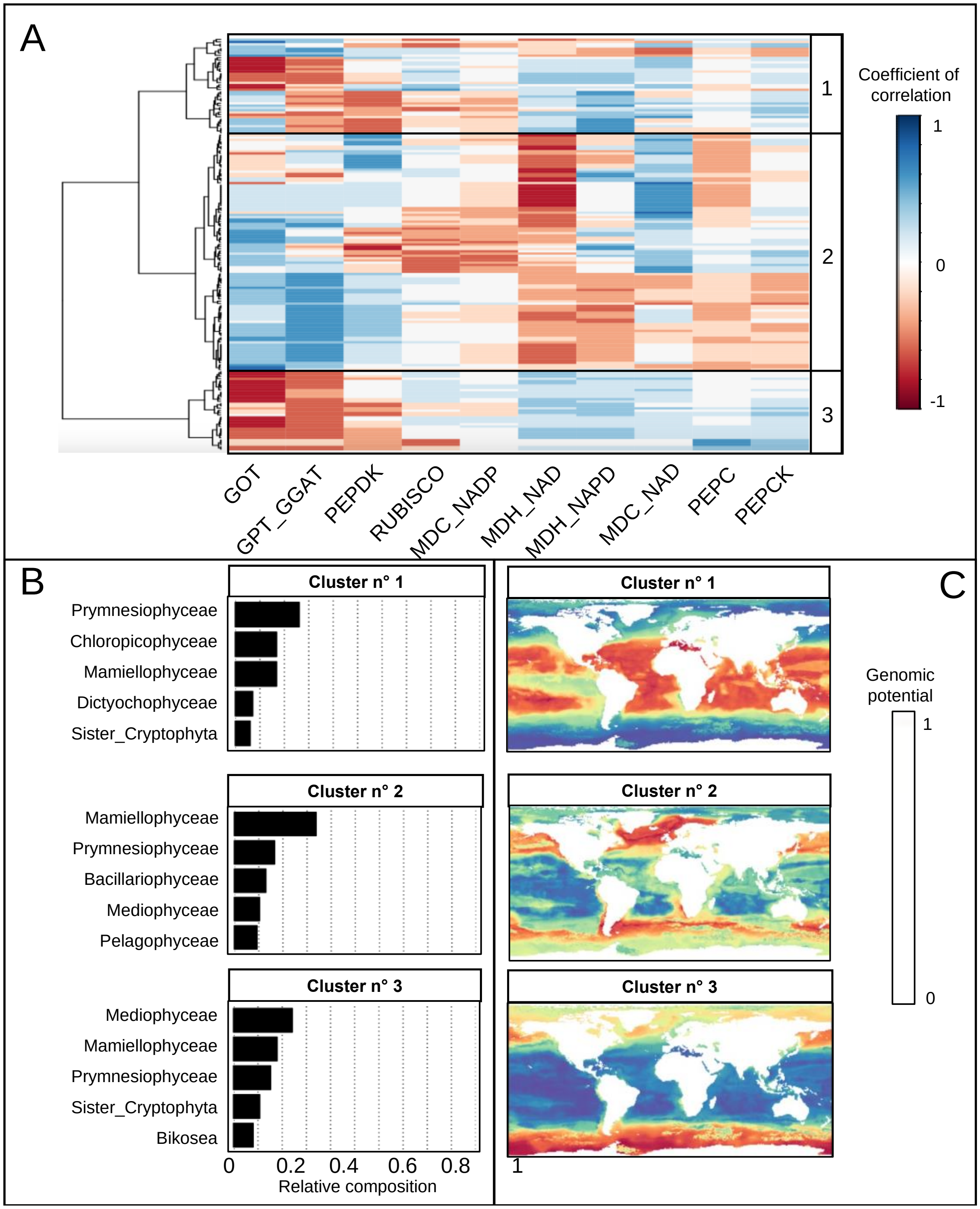

Fig. S6.

Estimated MAG-based taxonomic effect on standardized patterns with (A) the correlation between MAG distribution pattern clusters and projected genomic potential related to a given enzyme, (B) the taxonomic composition of each MAG cluster and (C) their corresponding projections.

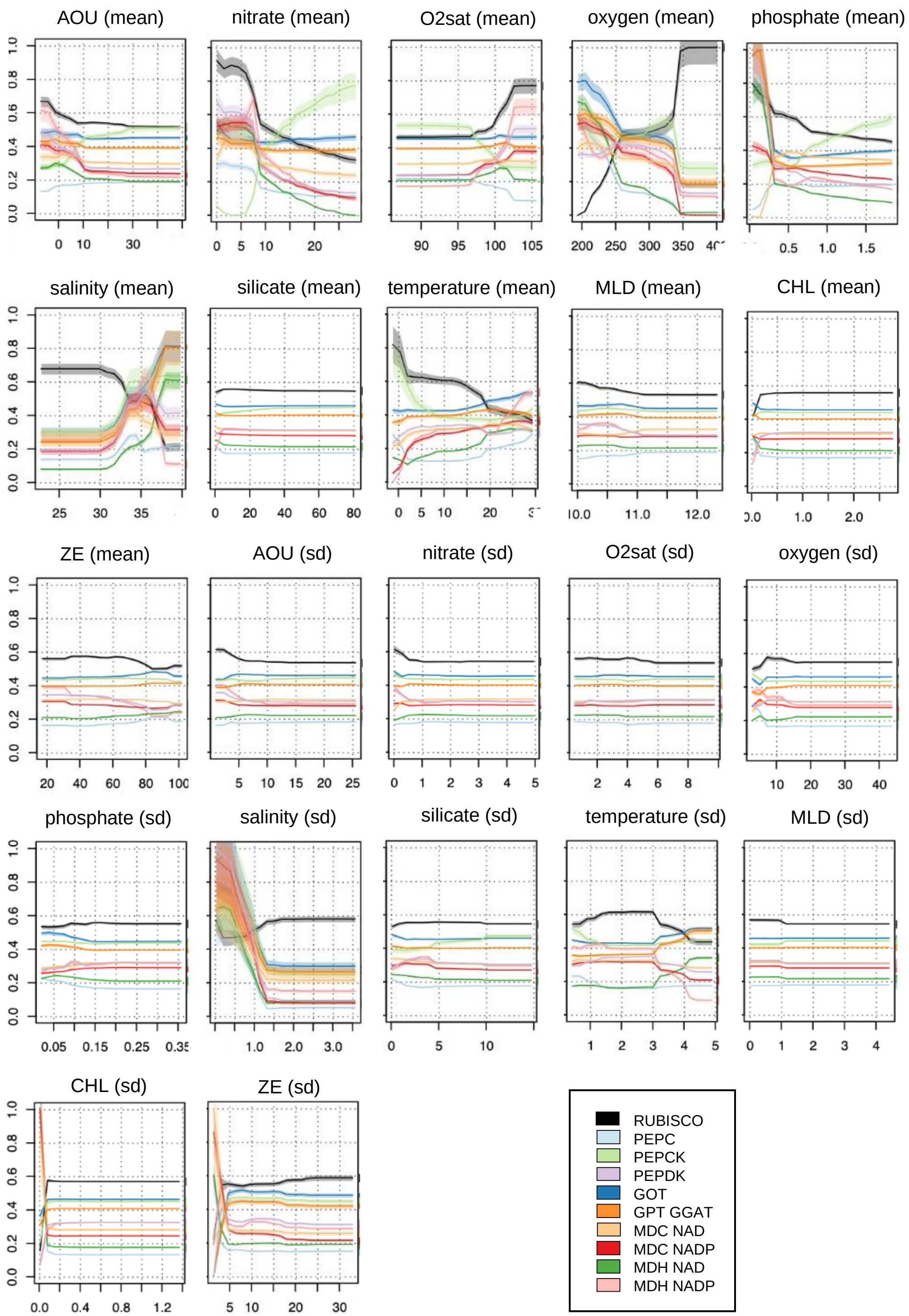

Fig. S7.

Partial dependence plots corresponding to the weighted pattern (i.e., re-scaled by the corresponding observed relative metagenomic reads abundance). The Y axis corresponds to the genomic potential while the X axis represents the environmental parameter possible values across world oceans. The full line and shading respectively correspond to the average response and standard deviation between bootstrap runs.

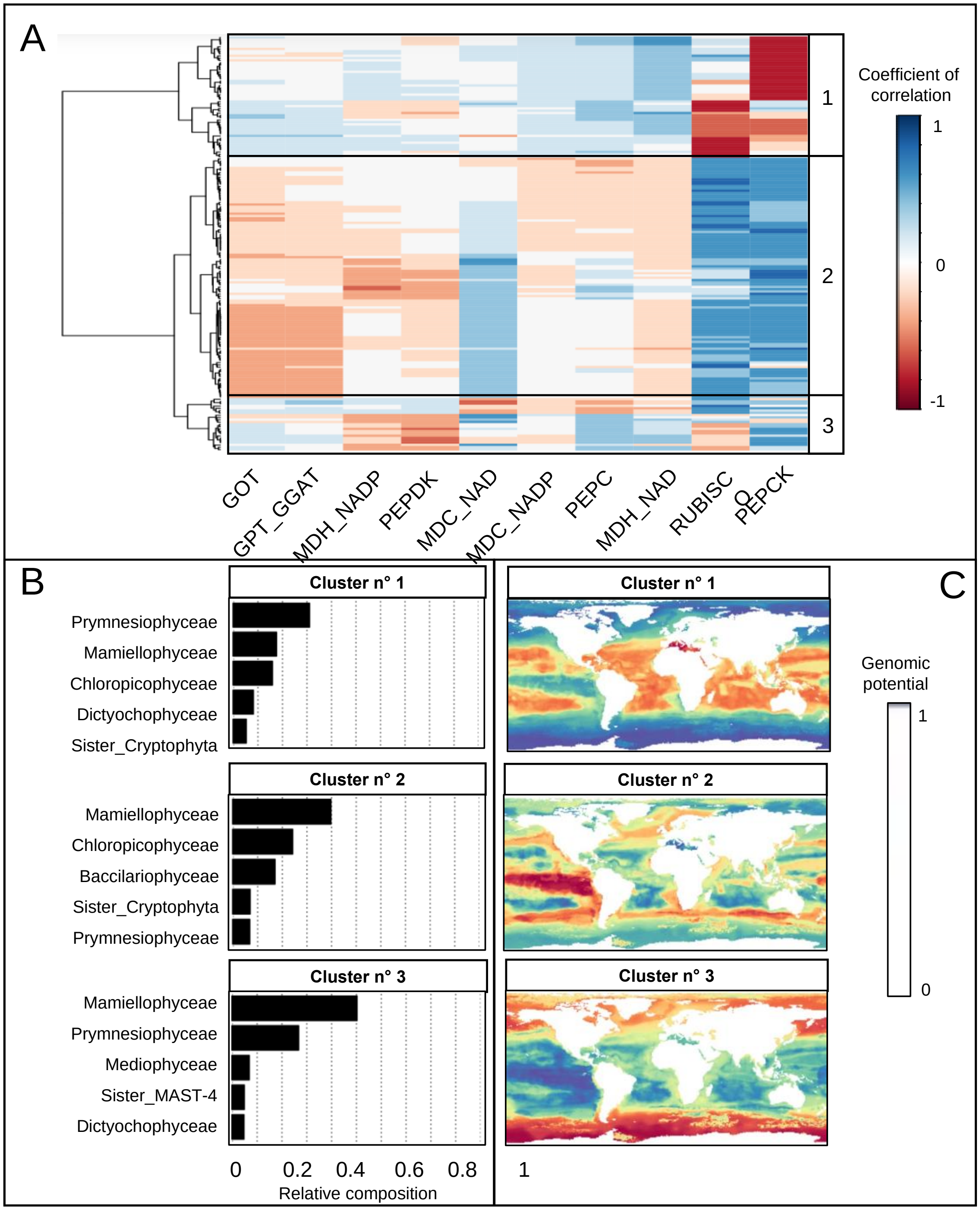

Fig. S8.

Estimated MAG-based taxonomic effect on weighted patterns (i.e., re-scaled by the corresponding observed relative metagenomic reads abundance) with (A) the correlation between MAG distribution pattern clusters and projected genomic potential related to a given enzyme, (B) the taxonomic composition of each MAG cluster and (C) their corresponding projections.

Table S1.

C4 carbon concentration-related enzymes and corresponding KEGG Orthology (KO) and enzyme (EC) reference annotations.

Enzyme	Description	KO	EC
PEPC	Phosphoenolpyruvate carboxylase	K01595	4.1.1.31
GOT	Aspartate aminotransferase, cytoplasmic	K14454	2.6.1.1
	Aspartate aminotransferase, mitochondrial	K14455	
PEPCK	Phosphoenolpyruvate carboxykinase (ATP)	K01610	4.1.1.49
MDH – NADP	Malate dehydrogenase (NADP)	K00051	1.1.1.82
MDH – NAD	Malate dehydrogenase (NAD)	K00024; K00025; K00026	1.1.1.37
MDC – NADP	Malate dehydrogenase (decarboxylating; NADP)	K00029	1.1.1.40
MDC – NAD	Malate dehydrogenase (decarboxylating; NAD)	K00028	1.1.1.39
GPT - GGAT	Alanine transaminase	K00814	2.6.1.2
	Glutamate – glyoxylate aminotransferase	K14272	2.6.1.4; 2.6.1.44
PEPDK	Pyruvate, orthophosphate dikinase	K01006	2.7.9.1
RUBISCO	Ribulose-1,5-biphosphate carboxylase oxygenase	K01601 K01602	4.1.1.39

Table S2.
Environmental climatologies considered in the model.

Name	Description	Reference
temperature	Sea surface temperature (°C)	Boyer et al. (56)
salinity	Sea surface salinity (unitless)	
oxygen	Dissolved Oxygen (µmol/kg)	
o2sat	Percent Oxygen Saturation (%)	
AOU	Apparent Oxygen Utilisation (µmol/kg)	
silicate	Silicate (µmol/kg)	
phosphate	Phosphate (µmol/kg)	
nitrate	Nitrate (µmol/kg)	
ZE	Depth of the Euphotic Zone (m)	Morel & Maritorena (57)
MLD	Mixed Layer Depth (m)	de Boyer Montégut et al. (58)

Data S1: Additional information on the Metagenome Assemble Genomes (MAGs) considered in this study. This supplementary data contains the data and metadata corresponding to each MAG. Associated quality checks including the horizontal coverage, completeness (%) and BUSCO completeness (%) of each MAG are also provided.
